# Mucormycosis (black fungus) in COVID-19 patients—Will it be another matter of concern in the midst of the COVID-19 flare-up in Bangladesh?

**DOI:** 10.5455/javar.2021.h524

**Published:** 2021-07-09

**Authors:** Md. Tanvir Rahman, Md. Golzar Hossain, A. M. M. Taufiquer Rahman, A. K. M. Moyeenul Huq, Shahnur Farzana, K. H. M. Nazmul Hussain Nazir

**Affiliations:** 1 Department of Microbiology and Hygiene, Faculty of Veterinary Science, Bangladesh Agricultural University, Mymensingh-2202, Bangladesh; 2 Naogaon District Hospital, Naogaon-6500, Bangladesh; 3 Department of Pharmacy, School of Medicine, University of Asia Pacific, Dhaka-1205, Bangladesh; 4 Department of Biochemistry, Community Based Medical College, Mymensingh-2200, Bangladesh

**Keywords:** Risk factors, mucormycosis, COVID-19, steroid

## Abstract

Many countries of the world have been combating the new variant of severe acute respiratory syndrome coronavirus 2. Black fungus is an opportunistic foe that may cause fatal infection in immunocompromised and steroid-treated coronavirus disease 2019 (COVID-19) patients. The COVID-19 associated mucormycosis (CAM) is now a serious concern throughout the world, including many Asian countries. Therefore, along with early and accurate diagnostic facilities, special care, and prompt, but coordinated approach are recommended to combat the CAM in patients.

Since the outbreak, coronavirus disease 2019 (COVID-19) has been grappling with Bangladesh’s health sector, social life, and economy. Bangladesh had faced the sudden second wave of COVID-19 with an increased number of daily new cases and death compared to the first wave. Recently, the more contagious delta variant of severe acute respiratory syndrome coronavirus 2 has been increasingly reported in Bangladesh with a higher death rate. As of July 2 2021, Bangladesh confirmed 930,042 COVID-19 cases and 14,778 deaths, having a 1.58% case fatality rate [1].

As the world is trying to cope with the devastating situation of COVID-19, another threatening condition named the “black fungus” has emerged around the globe, especially in India, the neighboring country of Bangladesh. In many Asian countries, COVID-19 associated mucormycosis (CAM) has been detected [2]. Black fungus, medically termed “Mucormycosis,” is a rare but deadly disease with a more than 50% mortality rate [3]. The disease has become more dangerous when affecting the COVID-19 patients. Mucormycosis is mainly associated with immunocompromised patients having poorly controlled diabetes conditions, in addition to long-term use of steroids for the treatment of COVID-19. Data confirmed that more than 90% of patients with CAM had diabetes throughout the world [4]. Clinical symptoms and pathogenesis of the CAM types include pulmonary mucormycosis, disseminated mucormycosis, rhino-orbital mucormycosis, etc. [5]. The relationship of COVID-19 and black fungus with various risk factors for the occurrence of mucormycosis is presented in Figure 1. Among reported CAM cases, the majority (71%) were from India, with 14,872 cases [6]. However, in Bangladesh, the number is very minimal, with a few cases; all of them had diabetes. Both Bangladesh and India share similar and higher occurrences of diabetes (8.1% *vs.* 8.9% of total adults), indicating that Bangladesh is also at higher risk for the outbreak of CAM [7].

**Figure 1.**
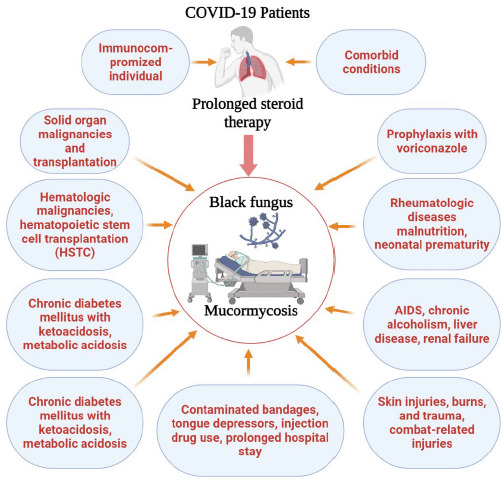
Relationship of COVID-19 and black fungus with the risk factors for mucormycosis.

Additionally, Mucorales are found in various environments, including soil and decaying organic waste such as compost piles, leaves, rotten wood, and animal excrement [8]. Due to their widespread presence in the environment, humans are constantly exposed to Mucorales. Therefore, good hygiene practice could also play a helpful role in preventing mucormycosis. Unfortunately, many people in Bangladesh (about 107 million) still deficit good hygiene at home [9]. Nonetheless, most importantly, diabetes might be a crucial risk factor for CAM due to the undiagnosed levels of diabetes in Bangladesh. In addition, people of Bangladesh are receiving steroids out of panic during COVID-19 infection, which may compromise their immune system and put them at higher risk of mucormycosis [10].

Although the situation of CAM in Bangladesh is not severe, the governmental health authorities should take appropriate initiatives to prevent untoward situations in the near future. It is now high time to focus on formulating guidelines for rapid diagnosis and management of mucormycosis in COVID-19 patients and building public awareness on CAM based on the analysis of the current situation at home and abroad. As the diabetes condition and use of steroids are directly associated with CAM, authorities should grow awareness within the populace on the risk factors and consequences of mucormycosis. Furthermore, antifungal drugs, mainly those commonly prescribed against mucormycosis such as Amphotericin B, should be made available homogenously throughout the country [11]. Although the health officials at the policy level have already announced that they are preparing guidelines for preventing and controlling mucormycosis infection, we recommend boosting their activities. Importantly, along with developing early and accurate diagnostic facilities, an adaptation of a more prompt coordinated approach such as treatment and management of immunocompromised COVID-19 patients is strongly recommended to prevent and combat the CAM.
